# Highly Specific and Effective Targeting of EGFRvIII-Positive Tumors with TandAb Antibodies

**DOI:** 10.3389/fonc.2017.00100

**Published:** 2017-05-19

**Authors:** Kristina Ellwanger, Uwe Reusch, Ivica Fucek, Stefan Knackmuss, Michael Weichel, Thorsten Gantke, Vera Molkenthin, Eugene A. Zhukovsky, Michael Tesar, Martin Treder

**Affiliations:** ^1^Affimed GmbH, Heidelberg, Germany; ^2^AbCheck s.r.o., Plzen, Czechia

**Keywords:** CD3, T cells, bispecific antibodies, tandem diabody, EGFRvIII, glioblastoma multiforme, affinity maturation, immunotherapy

## Abstract

To harness the cytotoxic capacity of immune cells for the treatment of solid tumors, we developed tetravalent, bispecific tandem diabody (TandAb) antibodies that recognize EGFRvIII, the deletion variant III of the epidermal growth factor receptor (EGFR), and CD3 on T-cells, thereby directing immune cells to eliminate EGFRvIII-positive tumor cells. Using phage display, we identified scFv antibodies selectively binding to EGFRvIII. These highly EGFRvIII-specific, fully human scFv were substantially improved by affinity maturation, achieving *K*_D_s in the picomolar range, and were used to construct a set of bispecific EGFRvIII-targeting TandAbs with a broad range of binding and cytotoxic properties. These antibodies exhibited an exquisite specificity for a distinguished epitope in the N-terminal portion of EGFRvIII, as shown on recombinant antigen in Western Blot, SPR, and ELISA, as well as on antigen-expressing cells in FACS assays, and did not bind to the wild-type EGFR. High-affinity EGFRvIII/CD3 TandAbs were most potent in killing assays, displaying cytotoxicity toward EGFRvIII-expressing CHO, F98 glioma, or human DK-MG cells with EC_50_ values in the range of 1–10 pM *in vitro*. They also demonstrated dose-dependent growth control *in vivo* in an EGFRvIII-positive subcutaneous xenograft tumor model. Together with the tumor-exclusive expression of EGFRvIII, the EGFRvIII/CD3 TandAbs’ high specificity and strictly target-dependent activation with no off-target activity provide an opportunity to target tumor cells and spare normal tissues, thereby reducing the side effects associated with other anti-EGFR therapies. In summary, EGFRvIII/CD3 TandAbs are highly attractive therapeutic antibody candidates for selective immunotherapy of EGFRvIII-positive tumors.

## Introduction

The human epidermal growth factor receptor (HER) family consists of four members: epidermal growth factor receptor (EGFR), HER2/neu, HER3, and HER4 (1, 2). In normal tissue, they are activated when specific ligands bind the extracellular domains (ECD) of EGFR, HER3, or HER4 leading to hetero- or homo-dimerization and subsequent activation of their kinase domains through conformational changes and intracellular tyrosine phosphorylation (3). The function of these receptors is dysregulated under pathophysiological conditions such as in colorectal cancer, head and neck cancer, lung cancer, and glioblastoma, and it is well established that this contributes to malignant transformation. Examples for dysregulation and aberrant signaling of the HER family of receptors include gene amplification and receptor overexpression, point mutations in the kinase domain resulting in ligand-independent constitutive kinase activation, or uncontrolled autocrine ligand stimulation (4). Specifically, treatment of head and neck, colorectal, or lung cancers targeting EGFR and HER2/neu has been clinically validated with the use of monoclonal antibodies (mAbs) including cetuximab (EGFR), panitumumab (EGFR), trastuzumab (HER2), or receptor tyrosine kinase inhibitors such as Tarceva (EGFR) or Iressa (EGFR) (5–7).

EGFRvIII is the most prevalent tumor-specific variant of EGFR. It is formed through an 801-bp deletion of exons 2–7 of the EGFR gene, entailing an in-frame deletion of 267 amino acids in the ECD and introduction of a single novel glycine residue at the newly formed junction (8, 9). This gene rearrangement deletes substantial portions of the ligand-binding domains and renders this truncated receptor ligand independent and constitutively active (10). While its expression has originally been described in up to 60% of glioblastoma multiforme (GBM) patients and numerous groups have found this tumor-specific mutation in other cancers such as squamous cell carcinoma of the head and neck (HNSCC), prostate, breast, or lung (NSCLC), EGFRvIII is not expressed in normal tissue, making it an ideal target for cancer therapy (11, 12).

Despite the clinical successes achieved with targeting the EGFR in colorectal carcinoma, HNSCC, or NSCLC, little therapeutic progress has been made in GBM and specifically targeting EGFRvIII (13–15). As such, there is still a high unmet medical need in GBM and several different therapeutic approaches have been tried or are currently in development; recent examples include Celldex peptide vaccination approach, rindopepimut in Phase 3, or its combination with an anti-VEGF antibody (bevacizumab) in Phase 2, or Amgen’s and Abbvie’s antibody–drug conjugate approaches with antibodies AMG 595 that completed Phase 1 and ABT-414 in Phase 2 development, respectively (16–18). Though these therapeutic approaches may offer a promise of more precise targeting compared to small molecule inhibitors, and initial results were very promising (19), they have not delivered breakthrough therapies: vaccination approaches have essentially been ineffective in large Phase 3 trials (20) and toxins conjugated to mAbs have either demonstrated off-target toxicity (21) or limited durability of response, with relapses in a high percentage of patients. In addition, CAR T-cell approaches using autologous T-cells engineered to express a chimeric antigen receptor targeting EGFRvIII have commenced clinical testing (22). Furthermore, the great therapeutic potential of bispecific antibody platforms has been recognized. Such compounds can be engineered either to target a tumor-specific antigen on target cells with one of their binding specificities and to recruit immune effector cells with the other specificity or to bind to two different antigens relevant for targeting aberrant pathways. Examples of bispecific antibody formats that are approved or in clinical development include bispecific T-cell engagers (BiTEs) (23) or tandem diabodies (TandAbs). TandAbs are composed of four variable domain fragments (Fv) of antibody heavy and light chains expressed as a single gene product connected by linkers. The translated polypeptide chains form the TandAb molecule by intermolecular head-to-tail dimerization (24). Importantly, unlike smaller bispecific antibodies, TandAbs provide two binding sites for each antigen, thereby maintaining the avidity of bivalent antibodies. TandAbs lack the Fc portion of classical antibodies but with their apparent molecular weight of >100 kDa exceeding the first-pass renal clearance threshold, offer a longer half-life and hence a pharmacokinetic advantage compared to smaller antibody constructs thus allowing flexibility of treatment regimens. The biodistribution of TandAbs, the ability to extravasate and also reach abluminal tumors is in agreement with predictions based on pharmacokinetic modeling and also confirmed by others (25). The TandAb platform is clinically validated and has shown potent cytotoxicity against target cells *in vitro* and *in vivo* (26–28).

In this study, we identified and successfully affinity matured highly EGFRvIII-selective, fully human scFv antibodies that are unique in their epitope specificity. Using the TandAb platform, we developed potent BiTEs for efficient immunotherapy of EGFRvIII-positive solid tumors. TandAb candidates were optimized regarding their target and effector cell-binding features and show excellent drug-like properties. Here, we describe their biophysical and functional *in vitro* and *in vivo* characterization and safety risk profiling.

## Materials and Methods

### Phage Display Screening

Proprietary phage display libraries of human scFv sequences were subjected to a preincubation on Nunc maxisorp surfaces coated with recombinant human EGFR-Fc. This preincubation step should deplete scFv from the libraries that bind to wild-type EGFR or to the Fc part of the fusion protein. To enrich EGFRvIII-Fc-specific binders from the depleted libraries, they were subjected to pannings with recombinant human EGFRvIII-Fc in two parallel approaches, with EGFRvIII-Fc coated to a plastic surface and with EGFRvIII-Fc captured *via* protein G beads (Dynabeads, Life Technologies) after incubation with the phage libraries in solution. Recovered phagemids were amplified in *E. coli*, packaged again into phage particles, and subjected to the next panning round. After two and three panning rounds, single colonies were picked, expression of soluble scFv was induced (29), and the bacterial extracts were screened for binding to EGFRvIII-Fc and EGFR-Fc in ELISA.

### Affinity Maturation

An AbAccel library design for the antibody framework pair IGHV5-51/IGLV3-25 was ordered from DistributedBio. The design included 52 randomized CDR positions, each with an individually defined distribution of amino acids. Variable heavy chain (V_H_) CDR3 of Li3G30 was retained. Three different loop lengths were built into Variable light chain (V_L_) CDR3. Gene fragments encoding the randomized positions of V_H_ and V_L_ were ordered from Geneart/Life Technologies and synthesized *via* TRIM technology. The fragments were cloned into the phage display vector pEXHAM (30), reaching a final library size of 3.7 × 10^8^ transformed *E. coli* cells. The library was packaged in phage particles and subjected to a panning and screening procedure to isolate variants with improved affinities and retained specificity for EGFRvIII. To assure that no EGFR-EGFRvIII cross-reacting or Fc-binding antibodies are selected in the process of affinity maturation, the library was depleted of EGFR binders by preincubation on EGFR-Fc prior to the first panning step. The panning was done with EGFRvIII-Fc antigen that was immobilized on a protein-binding plastic surface. To favor the enrichment of EGFRvIII-binding scFv with slow dissociation rates (*k*_d_) washing procedures included several incubation steps up to 30 min in duration. Another procedure was based on an overnight competition with soluble EGFRvIII. Moreover, the presence of soluble EGFR in the course of the panning procedure on immobilized EGFRvIII disfavored the enrichment of EGFR cross-reactive binders.

### Cell Lines and Cell Culture

DK-MG (ACC 277) and Jurkat (ACC 282) were purchased from DSMZ and cultured in RPMI 1640 medium supplemented with 10% heat-inactivated fetal calf serum (FCS), 100 U/mL Penicillin/Streptomycin, and 2 mM l-glutamine, herein referred to as complete RPMI medium (all from Life Technologies). F98 cells (CRL-2397), F98^EGFRvIII^ (CRL-2949), and F98^EGFR^ (CRL-2948) were purchased from ATCC, A431 cells were obtained from Dr. G. Moldenhauer (DKFZ Heidelberg), and cultured in DMEM supplemented with 10% FCS, 100 U/mL Penicillin/Streptomycin, and 2 mM l-glutamine. EGFRvIII- or EGFR-transfected F98 cell cultures were supplemented with 0.2 mg/mL Geneticin (G-418) to maintain stable antigen expression.

Flp-In CHO cells were purchased from Life Technologies and adapted to suspension growth in HyClone CDM4 CHO medium (GE Healthcare) supplemented with 2 mM l-glutamine, HT supplement, and 100 U/mL Penicillin/Streptomycin as well as 100 µg/mL Zeocin (all from Life Technologies) prior to transfection.

All cells were cultured under standard conditions at 37°C, 5% CO_2_, and subcultured according to standard protocols.

PBMCs were isolated from healthy volunteers’ buffy coat (German Red Cross, Mannheim, Germany) and T-cells enriched as previously described (26).

### Expression Constructs

Gene sequences encoding single-chain variable domain fragments (scFv), tetravalent bispecific TandAb antibodies, diabodies, tandem single-chain bispecific antibody (BiTE format), IgGs, and antigens were synthesized or subcloned using standard molecular biology techniques.

In scFv expression constructs, the variable heavy chain (V_H_) sequences and variable light chain sequences (V_L_) were joined by a flexible 18-aa linker (GGS)_6_. Bivalent diabodies targeting EGFRvIII were constructed with a domain order ${\text{V}}_{\text{H}}^{\text{EGFRvIII}}-{\text{V}}_{L}^{\text{EGFRvIII}}$ separated by a 6-aa linker sequence (GGS)_2_.

Tetravalent bispecific EGFRvIII- and CD3-targeting TandAb antibodies were designed with different target- and effector cell-binding domains, connected with linker sequences. The standard domain order and linker setting for TandAbs was as follows: ${\text{V}}_{\text{L}}^{\text{CD3}}-{\left(\text{GGS}\right)}_{\text{2}}-{\text{V}}_{\text{H}}^{\text{EGFRvIII}}-{\left(\text{GGS}\right)}_{\text{2}}-{\text{V}}_{\text{L}}^{\text{EGFRvIII}}-{\left(\text{GGS}\right)}_{\text{2}}-{\text{V}}_{\text{H}}^{\text{CD3}}.$ Some additional TandAb variants containing (GGS)_3_- or (GGS)_4_-linkers between V_L_ and V_H_ segments were constructed. Anti-CD3 sequence clone LcHC21ktay (26) or humanized anti-human and cynomolgus CD3 (CD3_x_) variants differing in their affinities for CD3 (28) were used in TandAbs for T-cell engagement. Gene sequences of published EGFRvIII-targeting comparator antibodies MR1-1 × OKT3 tandem single-chain bispecific antibody (31–33) and ch806 (34) were synthesized (Geneart). All antibody fragments were research derivatives containing at the C-terminus an AAAGS linker and (His)_6_-tag to facilitate purification and detection. The sequences encoding human EGFR, EGFRvIII, as well as a truncated form of EGFRvIII lacking the six N-terminal amino acids _1_LEEKKG_6_ were synthesized (Geneart) and used for the generation of stably transfected cells overexpressing these receptor variants on the cell surface. For the expression of soluble antigen variants, the ECD sequences of EGFR, EGFRvIII, truncated EGFRvIII, and CD16A were fused to the Fc-portion of human IgG1. Through intramolecular disulfide bond formation in the hinge region of the Fc-portion, these antigen fusions form covalent dimers of two identical chains. To facilitate recombinant protein secretion, all expression constructs were designed to contain coding sequences for an N-terminal signal peptide and were subcloned into the mammalian expression vector pcDNA5/FRT to facilitate stable cell generation and expression of the products in Flp-In CHO cells. Sequences of all constructs were confirmed by DNA sequencing.

### Expression, Purification, and Biochemical Analysis of Recombinant Antibodies and Antigens

Stable, recombinant protein-expressing cells were generated and used for protein expression as previously described (28). Protein expression titers and product integrity in cell culture supernatants (CCS) were analyzed by SDS-PAGE using Criterion Stain-Free technology (Bio-Rad). Product titers were determined semi-quantitatively by comparison with a reference protein of known concentration. His-tagged antibody fragments and TandAb candidates were purified from the CCS by HisTrap FF 5 mL column (GE Healthcare) chromatography followed by preparative size exclusion chromatography (SEC) on Superdex 200 (GE Healthcare). Purified proteins were buffer exchanged into 10 mM Na-acetate pH 5 using PD-10 desalting columns (GE Healthcare), concentrated by centrifugational ultrafiltration using Amicon Ultra 15, MWCO 10 kDa (Millipore) typically to a concentration of 1.0–1.5 mg/mL, and stored at −80°C. Protein concentration was determined by UV spectroscopy (280 nm). IgG and Fc-fusion antigens were purified *via* protein A.

CD3ε antigen was purchased from Creative BioMart.

### Measurement of Binding by SPR

All experiments were performed on a Biacore T200 System (GE Healthcare) at 25°C. For dilution of samples, running buffer HBS-P+ was used. Anti-human Fc IgG C1-chip was prepared by covalent amine coupling using human Antibody capture Kit (GE Healthcare) and Amine-Coupling-Kit. A target level between 100 and 310 RU for all 4 flow cells (fc) was reached.

EGFRvIII-Fc and EGFR-Fc fusion proteins (diluted to 6.25 nM) were captured on the chip at a flow rate of 5 µL/min (EGFRvIII-Fc in fc2 50 s, EGFRwt-Fc in fc4 50 s). Samples were measured in Multi Cycle Kinetic runs (60 µL/min, contact time 180 s, dissociation time 540 s, Flow cells fc 1, 2, 3, 4) with two start-up cycles prior to measurements. Data were double referenced by signals in flow cells fc2-1, fc4-3 and using a zero concentration buffer sample. Chip was regenerated using 3 M MgCl_2_ (10 µL/min, 30 s, fc 1, 2, 3, 4). Data were fitted to 1:1 Binding model (Rmax locally fitted).

For peptide competition assays, EGFRvIII-Fc antigen was captured on an anti-human IgG CM5 chip, different concentrations of the EGFRvIII-specific fusion peptide PEPvIII (Genescript) were mixed with a fixed concentration of 100 nM of anti-EGFRvIII diabody and binding signals were analyzed on the captured EGFRvIII.

### SDS-PAGE and Western Blot

To analyze binding specificity and epitope recognition of different EGFR- and EGFRvIII-binding antibodies, SDS-PAGE and Western Blot analyses were performed. Equal amounts of the purified EGFR-Fc or EGFRvIII-Fc antigen variants were mixed with non-reducing or reducing 2× SDS-PAGE sample buffer containing dithiothreitol (DTT) as reducing agent. Samples with DTT were heated at 95°C for 5 min prior to loading on 4–20% Criterion TGX Precast SDS-PAGE Gels (Bio-Rad). SDS-PAGE was run in 1× Tris/Glycine/SDS buffer (Bio-Rad) at 300 V for ~25 min. Total protein was visualized using the Criterion Stain-free Molecular Imaging System (Bio-Rad). Page Ruler Unstained Protein ladder (Thermo Scientific) was used as a molecular weight marker. Protein was blotted on PVDF membranes using Semi-dry blotting Fastblot system (Bio-Rad). Membranes were blocked in 3% (w/v) skim milk powder in 1× TBS for 30 min at room temperature. Primary antibodies were diluted to 2 µg/mL in 3% (w/v) skim milk powder in 1× TBS and incubated with individual membrane pieces for 1 h on a shaking platform. Membranes were washed with TBS + 0.1% (v/v) Tween 20 three times for 10 min each and once with TBS prior to incubation with secondary HRP-conjugated detection antibodies, anti-Penta-HIS-HRP (QIAGEN) for the detection of the bound His-tagged antibody constructs, 1:5,000 diluted in 3% skim milk powder in TBS for 1 h on a shaking platform. Membranes were washed as before and HRP-mediated color development on the membrane was initiated by the addition of 0.06% DAB + 0.02% CoCl_2_ + 0.015% H_2_O_2_ in TBS. After signal development, membranes were washed with water, dried, and scanned.

### Epitope Mapping

To reconstruct epitopes of the EGFRvIII ECD, a library of 15-mer peptides was synthesized. In addition to peptides covering the native amino acid sequence of EGFRvIII, amino acid substitutions were introduced into all relevant positions to pinpoint crucial binding residues. An amino-functionalized polypropylene support was obtained by grafting with a proprietary hydrophilic polymer formulation, followed by reaction with t-butyloxycarbonyl-hexamethylenediamine using dicyclohexylcarbodiimide with N-hydroxybenzotriazole and subsequent cleavage of the Boc groups using trifluoroacetic acid. Standard Fmoc-peptide synthesis was used to synthesize peptides on the amino-functionalized solid support by custom modified JANUS liquid handling stations (Perkin Elmer). The binding of antibody to each of the synthesized peptides was tested by ELISA. The peptide arrays were incubated with primary antibody solution (overnight at 4°C). After washing, the peptide arrays were incubated with a 1:1,000 dilution of an appropriate antibody peroxidase conjugate for 1 h at 25°C. After washing, the peroxidase substrate 2,2′-azino-di-3-ethylbenzthiazoline sulfonate and 2 µL/mL of 3% H_2_O_2_ were added. After 1 h, the color development was measured. The color development was quantified with a charge-coupled device-camera and an image processing system.

### ELISA

The 96-well ELISA plates (Immuno MaxiSorp; Nunc) were coated overnight at 4°C with recombinant antigen in 100 mM Carbonate–bicarbonate buffer. EGFRvIII-Fc antigen variants were coated at 4 µg/mL, EGFR-Fc at 6 µg/mL, or CD3ε antigen at 0.75 µg/mL. After a blocking step with 3% (w/v) skim milk powder (Merck) dissolved in PBS, serial dilutions of different antibodies in PBS containing 0.3% (w/v) skim milk powder were incubated on the plates for 1.5 h at room temperature. After washing three times with 300 µL/well of PBS containing 0.1% (v/v) Tween 20, plates were incubated with secondary detection conjugates for 1 h at room temperature. For the detection of bound His-tagged antibody analytes, Penta-HIS-HRP (Qiagen) (1:3,000 dilution), for the detection of other control antibodies, anti-kappa LC-HRP (Sigma), Protein L-HRP (Thermo Scientific), or HRP-conjugated anti-mouse IgG (Dianova) were used as secondary detection reagents followed by washing and tetramethylbenzidine substrate reaction (Seramun). The reaction was stopped after ~2 min through the addition of 100 µL/well of 0.5 M H_2_SO_4_. The absorbance was measured at 450 nm using a multilabel plate reader (Victor, Perkin Elmer). Absorbance values were plotted and analyzed using non-linear regression, sigmoidal dose–response (variable slope), least squares (ordinary) fit with GraphPad Prism version 6.07 (GraphPad Software, La Jolla, CA, USA). For the calculation of *K*_D_ values, non-linear regression was performed with the equation one site binding (hyperbola) and least squares (ordinary) fit using GraphPad Prism version 6.07.

### Determination of Antibody Binding to Cells by Flow Cytometry

Binding on cells of different antibodies was analyzed by flow cytometry as previously described (26).

### Cell Surface Retention Assay

To quantify the retention of EGFRvIII/CD3 TandAbs on EGFRvIII-positive cells, aliquots of CHO^EGFRvIII^ cells were incubated with 10 µg/mL of the TandAbs for 45 min on ice. After repeated washing with ice-cold FACS buffer [PBS (Life Technologies) containing 2% heat-inactivated FCS (Life Technologies), and 0.1% sodium azide (Roth)], aliquots of 1 × 10^6^ cells were suspended in 10-mL FACS buffer supplemented with 10% heat-inactivated FCS at 37°C for the indicated time periods to allow dissociation. After repeated washing, cell-retained TandAbs were detected by staining of the cells on ice with mAb anti-His clone 13/45/31/2 (Dianova, Hamburg, Germany) followed by FITC-conjugated goat anti-mouse IgG (Dianova) and flow cytometric analysis. The mean fluorescence values at time-point 0 were taken to be 100%, and the percentage of remaining TandAb was analyzed by non-linear regression using GraphPad Prism.

### Assessment of Target Cell-Killing Mediated by EGFRvIII/CD3 TandAb Antibodies in Cytotoxicity Assay

T-cells or PBMCs used as effector cells were characterized by flow cytometry as described (26). Killing of CHO cells stably transfected to overexpress EGFRvIII (CHO^EGFRvIII^), EGFR (CHO^EGFR^), or untransfected CHO cells (CHO) was analyzed in FACS-based cytotoxicity assays. The cell lines were generated as previously described and were cultured under standard conditions. For the cytotoxicity assay, target cells were harvested, washed twice with RPMI 1640 medium without FCS, and resuspended in diluent C provided in the PKH67 Green Fluorescent Cell Linker Midi Kit to a density of 2 × 10^7^/mL. The cell suspension was then mixed with the equal volume of a double-concentrated PKH67-labeling solution (e.g., 1 µL PKH67 in 250 µL diluent C) and incubated for 2–5 min at room temperature with periodical mixing according to the manufacturer’s instructions. The staining reaction was stopped by adding the equal volume of FCS and incubation for 1 min. After washing the labeled target cells with complete RPMI medium, cells were counted and resuspended to a density of 2 × 10^5^/mL in complete RPMI medium. About 2 × 10^4^ target cells were then seeded together with T-cells at an E:T ratio of 5:1 and the indicated antibodies in individual wells of a round-bottom 96-well microplate in a total volume of 200 μL/well. Usually nine serial 1:5 dilutions starting at 30 µg/mL were tested. Spontaneous cell death and killing of targets by effectors in the absence of antibodies were determined in at least three replicates on each plate. TandAb-mediated killing was usually determined in duplicates. The assay plates were incubated for 40–48 h at 37°C in a humidified atmosphere with 5% CO_2_. After incubation, cultures were washed once with FACS buffer and resuspended in 150-µL FACS buffer supplemented with 2-µg/mL PI. The absolute amount of living target cells characterized by a positive green PKH67 staining and negative for the PI staining was measured using a Millipore Guava EasyCyte flow cytometer (Merck Millipore). Based on the measured remaining living target cells, the percentage of specific cell lysis was calculated according to the following formula: [1 − (number of living targets_(sample)_)/(number of living targets_(spontaneous)_)] × 100%. The lysis values obtained for a given antibody concentration were determined in duplicates and analyzed by sigmoidal dose–response/four parameter logistic fit analysis using Graphpad Prism software version 6.07 and used to calculate EC_50_ values, mean, and SD of replicates of percentage of lysis.

Killing of DK-MG cells, F98^EGFRvIII^, F98^EGFR^, or F98 cells was analyzed in calcein-release assays as previously described (26).

### Assessment of Cell Proliferation in PBMC Cultures or T-Cell Activation in the Presence of EGFRvIII/CD3 TandAb Antibodies

To assess whether EGFRvIII/CD3 TandAb-induced activation of T-cells is dependent on the presence of target cells, primary human PBMC (4 × 10^5^/well) were cultured in the presence of increasing concentrations EGFRvIII/CD3 TandAbs in the absence of EGFRvIII-positive target cells. After 5 days incubation, proliferation was assessed in a BrdU incorporation assay as previously described (28).

T-cell activation marker induction by EGFRvIII/CD3 TandAb was analyzed by FACS: primary human T-cells were cultured for 24 h with or without 10 µg/mL of TandAb in the presence or absence of F98^EGFRvIII^ target cells at an E:T ratio of 1:1 before T-cells were analyzed by flow cytometry for expression of the activation markers CD25, CD137, and CD69 after staining of the cell cultures with CD4-FITC/CD8-FITC (Beckman-Coulter) in combination with CD25-PE (Beckman-Coulter), CD137-APC (BD Biosciences), or CD69-APC-Cy7 (BD Biosciences). As a control, cells were cultured in the absence of antibodies and in the absence of target cells.

### *In Vivo* F98^EGFRvIII^ Xenograft Tumor Model

An appropriate number of experimental groups of immunodeficient NOD/scid mice (NOD/MrkBomTac-Prkdcscid, Taconic Denmark) (*n* = 8 mice per group) were xenotransplanted by subcutaneous injection with a suspension of 4 × 10^6^ of F98^EGFRvIII^ cells mixed with 1 × 10^7^ purified human PBMC from healthy donors on day 0. To account for potential donor variability of the PBMCs, each of the experimental groups was subdivided into two cohorts (*n* = 4 mice per cohort) each receiving PBMCs of one individual donor only. Animals were dosed intravenously into the tail vein 2 h post-tumor cell inoculation and subsequently after 24, 48, 72, and 96 h with the respective doses as indicated in the figures of the TandAb test items. Control groups received vehicle only. An additional control group was dosed intraperitoneal twice a week with 1 mg of cetuximab (Erbitux) starting on day 0. The tumor volume was monitored three times per week from day 3 by measuring the large diameter and the small diameter of a tumor with a caliper. Tumor volumes were calculated from diameters according to the following formula: Vol. = (small diameter)^2^ × large diameter × 0.5.

All animal experiments were performed at the Heidelberg Pharma GmbH animal facility. The study and the protocol were reviewed and approved by Heidelberg Pharma’s “Tierschutzausschuss” (ethical committee) to guarantee ethical standards which are according to the German animal welfare law based on EU-regulation (2010/63 EU). Accreditation of tumor studies was done by the mid-level local authority (Regierungspräsidium Karlsruhe), supervised by the lower-level local authority (Veterinäramt Wiesloch), regulated by European Union law and directives as well as German national law.

## Results

### Isolation and Affinity Maturation of Highly EGFRvIII-Specific scFv Antibodies

#### Discovery of Li3G30

EGFRvIII-specific single-chain variable fragment antibodies (scFv) were isolated from a phage display library with a total complexity of 10^10^. The library consisted of separate parts, which differed in the source of diversity and which were kept separately during the selections. All EGFRvIII-specific scFv were isolated from the part of the library, that was cloned from donor-derived V_H_ and V_L_ domains, featuring IgM-specific priming for the amplification of V_H_ encoding sequences as described previously (35, 36). The selection strategy to enrich EGFRvIII-specific binders during the phage panning process (37) implemented counter-selection steps to repress the enrichment of antibodies that recognize the wild-type EGFR. In particular, binders to wild-type EGFR were removed from the library in a depletion step prior to the panning with EGFRvIII. Two parallel selection strategies were applied with EGFRvIII-Fc being either immobilized on a plastic surface (solid phase panning) or captured by Protein G beads after incubation with the phage library in solution (solution panning). Soluble scFv expressed by a total of 1,056 individual clones after 2 and 3 panning rounds were screened in ELISA for binding to EGFRvIII-Fc and EGFR-Fc. The majority of scFv turned out to recognize both forms of the protein. However, two individual sequences with specific binding to EGFRvIII-Fc were identified from the panning in solution, and one further sequence was identified from the solid phase panning approach. The EGFRvIII-specific binding without background on wild-type EGFR was confirmed in a second ELISA and flow cytometry with transfected cells expressing either EGFRvIII or EGFR (data not shown). Out of these *de novo* identified EGFRvIII-specific binders, clone Li3G30 showed highly specific but low-affinity binding to EGFRvIII-Fc antigen as measured by SPR with a *K*_D_ value of 17.5 nM (Table 1). To improve target-binding affinities and reduce dissociation, affinity maturation of the EGFRvIII-specific binders was pursued.

**Table 1:** **EGFRvIII-specific antigen-binding constants of monovalent scFv and bivalent tandem diabody (TandAb) antibody variants containing *de novo* identified (parental) or affinity matured EGFRvIII-binding Fv sequences measured by SPR**.

EGFRvIII-binding Fv sequence		scFv			EGFRvIII/CD3_x_ TandAbs		
		*K*_D_ (nM)	*k*_a_ (1/Ms)	*k*_d_ (1/s)	*K*_D_ (nM)	*k*_a_ (1/Ms)	*k*_d_ (1/s)
Parental	Li3G30	17.5	1.50E+5	2.63E−3	0.567	8.70E+5	4.93E−4
Affinity matured	A3	0.25	1.43E+6	3.57E−4	0.006	3.43E+6	2.08E−5
	A4	0.16	3.53E+6	5.49E−4	0.011	8.22E+6	8.79E−5
	A6	0.85	9.45E+5	8.04E−4	0.043	2.31E+6	9.81E−5
	A7	0.80	2.85E+6	2.30E−3	0.024	4.54E+6	1.08E−4
	A8	6.50	1.55E+6	1.01E−2	0.095	1.90E+6	1.80E−4
	A9	2.11	1.35E+6	2.86E−3	0.045	1.44E+6	1.56E−4

*Sensorgrams and fits of SPR measurements are summarized in Figure S2 in Supplementary Material for TandAb candidates*.

#### Affinity Maturation

Affinity maturation of anti-EGFRvIII sequences was performed based on a computationally designed library, which reflects the naturally occurring diversity of amino acids in CDR positions (38). To maximize the chance of preserving the specificity during affinity maturation, the CDR-H3 sequence of the parental scFv was retained. The positional frequencies of amino acids in each position of the remaining CDRs was tailored to the germline genes IGHV5-51 and IGLV3-25, which were found to be the closest compared to the sequence of the parental antibody Li3G30. Framework positions were kept constant and identical to the germline encoded sequence in the library. High-affinity EGFRvIII-specific scFv were successfully enriched during the phage panning and identified by ELISA screening (data not shown). Selected scFv were further tested for binding to EGFRvIII-expressing CHO and F98 cells in flow cytometry. ScFv binding to EGFR-expressing and/or untransfected cells were excluded from further analyses. An initial ranking based on binding constants measured in SPR was used to identify scFv with improved binding affinities to the EGFRvIII antigen compared to the parental clone Li3G30. Two of the tested EGFRvIII-specific domains, A3 and A4 had >50-fold improved *K*_D_ values relative to the parental scFv (Li3G30) and 4-fold or higher improvement in the dissociation rate (*k*_d_) (Table 1). The best variants, A3, A4, A6, A7, A8, and A9, were selected for the construction of bivalently binding diabodies and tetravalent bispecific EGFRvIII/CD3 TandAbs and characterized in detailed SPR measurements (Table 1).

### Reformatting of Binding Domains, Construction and Expression of Multivalent and Bispecific Antibody Formats

**Figure 1:**
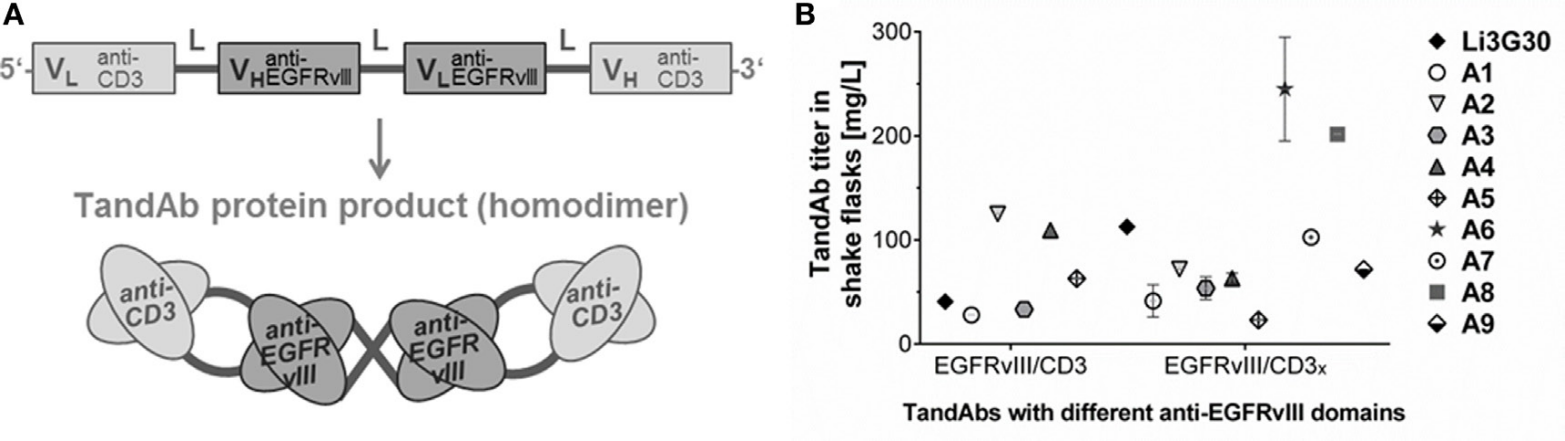
**Design, production, and purification of recombinant EGFRvIII-targeting tandem diabodies (TandAbs)**. **(A)** Scheme of the gene design, domain order, and postulated folding by dimerization of tetravalent bispecific EGFRvIII/CD3 TandAbs. The gene arrangement is shown from 5′ to 3′-end encoding the monomeric polypeptide. N-terminal signal peptide and C-terminal His tag are not shown. **(B)** Expressability of different classes of EGFRvIII-targeting TandAbs containing different T-cell-binding moieties, CD3 (human CD3-specific), or CD3_x_ (human/cynomolgus cross-reactive), and EGFRvIII-binding moieties was analyzed at research stage in shake flasks. For candidates expressed more than once, mean titers are plotted with error bars indicating SDs.

The affinity matured EGFRvIII-binding Fv sequences and the low-affinity (parental) domain, Li3G30, were used for the generation of different multivalent antibody domain fusion formats (39): bivalently binding anti-EGFRvIII diabodies, and EGFRvIII/CD3 TandAbs. In TandAbs, the EGFRvIII-binding domains were combined with human/cynomolgus cross-reactive anti-CD3 (CD3_x_) or human CD3-specific (CD3) domains capable of recruiting T-cells as immune effector cells (28). A domain order with alternating variable light chain and heavy chain segments connected by linker sequences, resulting in the formation of a dimeric TandAb with two binding sites for EGFRvIII in the core and two binding sites for CD3 in the external positions of the TandAb product was chosen (Figure 1A). Diabodies and TandAb candidates were produced using CHO-based expression in shake flasks as previously described (26). Production cultures showed good growth and viability and the TandAbs represented the main protein product in the cell CCS harvested after 10 days (Figure S1A in Supplementary Material; data not shown). Expression titers of different TandAb candidates containing various domain combinations, each expressed from a single gene copy integrated into the CHO genome ranged from 25 to ~250 mg/L (Figure 1B), allowing the production and purification of sufficient amount and quality of material for subsequent characterization *in vitro* and *in vivo*. TandAbs containing His tags were purified *via* immobilized metal ion affinity chromatography followed by preparative SEC resulting in highly pure homodimeric TandAb (typically >95%) as demonstrated by analytical SEC (Figure S1B in Supplementary Material). Integrity of the purified proteins was also assayed by SDS-PAGE, whereby under denaturing, reducing, and non-reducing conditions, TandAbs run as monomeric polypeptides (data not shown). Purified EGFRvIII/CD3 TandAbs exhibited good stability under accelerated conditions, tested for up to 7 days in a standard buffer, as exemplarily shown for the EGFRvIII^A6^/CD3_x_ TandAb (Figure S1C in Supplementary Material). EGFRvIII/CD3 TandAbs appeared as the main product in form of the intact homodimer under all conditions with a retention time in the SEC analysis consistent with an apparent molecular mass of ~105 kDa.

### EGFRvIII-Targeting Antibody Formats Bind to an EGFRvIII-Specific Epitope with High Affinity and Selectivity

Apparent EGFRvIII-binding affinities in the tetravalent bispecific TandAbs were more than 10-fold improved relative to the monovalently binding scFv. The highest anti-EGFRvIII affinity, a *K*_D_ of 6 pM was achieved for the EGFRvIII^A3^/CD3_x_ TandAb (Table 1; Figure S2 in Supplementary Material). Reduced dissociation rates were achieved by both, affinity maturation and reformatting into bivalently binding TandAbs (Table 1) or diabodies (data not shown).

**Figure 2:**
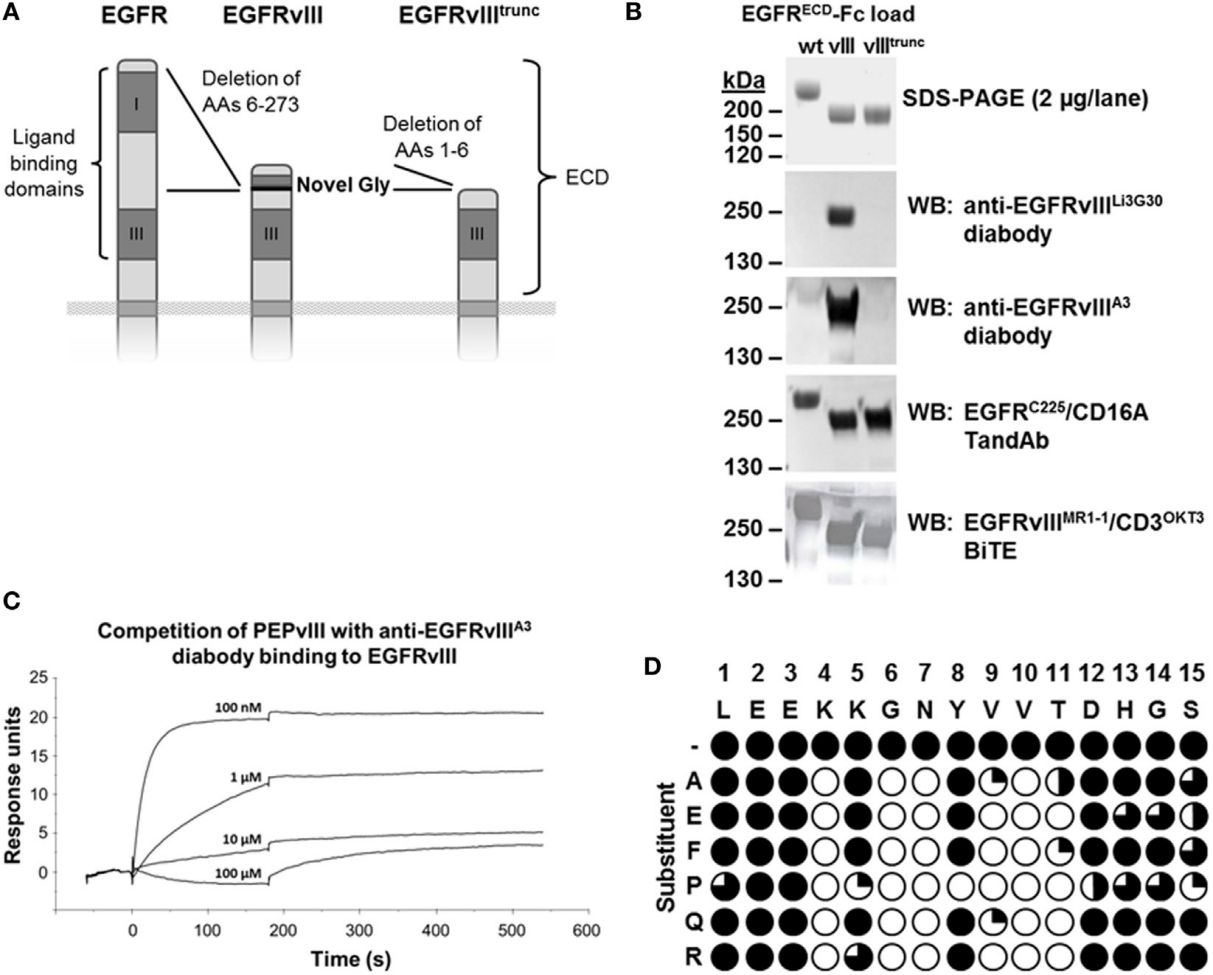
**Binding specificity for EGFRvIII antigen**. **(A)** Scheme of the extracellular domains (ECD) of EGFR, EGFRvIII, and a further truncated EGFRvIII variant EGFRvIII^trunc^ (recombinant, not naturally occurring). **(B)** Binding of different EGFRvIII- or EGFR-targeting antibodies or antibody fragments to recombinant Fc-fusions of these ECD variants after non-reducing SDS-PAGE and Western Blot. EGFRvIII^ECD^ due to the 267 amino acid deletion has an ~25 kDa smaller molecular size than EGFR^ECD^, resulting in an ~50 kDa size difference in the dimeric form of the Fc-fusion proteins under non-reducing conditions. **(C)** Binding of the anti-EGFRvIII^A3^ diabody to immobilized EGFRvIII antigen is efficiently outcompeted with increasing concentrations of EGFRvIII-specific peptide PEPvIII (aa-sequence: LEEKKGNYVVTDH). **(D)** Normalized ELISA signals for binding of anti-EGFRvIII^A3^ diabody to EGFRvIII N-terminal epitope spanning 15-mer peptides containing single amino acid substitutions in each position of the native sequence. Filled circles indicate strong binding to the native sequence or correspondingly substituted sequences, open circles indicate positions in which substitutions completely disrupted binding.

In addition to SPR measurements, binding to different antigen variants, EGFR, EGFRvIII, and an artificial, further truncated form of EGFRvIII (EGFRvIII^trunc^) (Figure 2A) was analyzed after SDS-PAGE and Western Blot (Figure 2B). Binding of our high- and low-affinity EGFRvIII-specific diabodies was clearly dependent on the presence of the N-terminal EGFRvIII-specific neoepitope (9). Truncation of the six N-terminal amino acids LEEKKG in EGFRvIII (of which Glycine in position 6 is unique to EGFRvIII, introduced at the junction during in-frame deletion of exons 2–7 of the EGFR gene) (Figure 2A) abolished binding after SDS-PAGE and Western Blot (Figure 2B). A control TandAb construct containing cetuximab-derived anti-EGFR Fv sequences (C225) detected all three antigen variants under non-reducing conditions confirming the conformational integrity of the extracellular domain III that is a prerequisite for cetuximab’s binding (40). In contrast to our EGFRvIII-specific diabodies, a reproduction of the published EGFRvIII^MR1-1^/CD3^OKT3^ bispecific antibody (33, 41) recognized all three EGFR antigen variants after SDS-PAGE and Western Blot, indicating that binding of this BiTE is not as strictly specific for EGFRvIII (Figure 2B). Reactivity of all antibodies was maintained if the antigen samples were run under reducing conditions, except for cetuximab which is known to recognize a discontinuous conformational epitope (40) (data not shown).

The requirement of the EGFRvIII-specific neoepitope sequence for binding of our antibodies was also confirmed in peptide competition experiments in SPR, where binding of an anti-EGFRvIII diabody containing the affinity matured domain A3, to EGFRvIII-Fc could be efficiently and concentration-dependently outcompeted with a linear peptide containing the EGFRvIII-specific fusion sequence LEEKKGNYVVTDH (PEPvIII) (Figure 2C).

To clarify in detail which amino acids of the EGFRvIII-specific neoepitope are recognized by our EGFRvIII-binding domains, linear peptide epitope mapping was performed. Different sets of linear 15-mer peptides were tested for recognition by our anti-EGFRvIII bivalently binding diabody, containing the affinity matured domain A3 or the parental domain Li3G30 (Figure 2D; data not shown). Binding of the diabodies was only measureable on peptides containing the intact N-terminal EGFRvIII neoepitope. Peptides lacking up to three N-terminal amino acids of EGFRvIII (_1_L_2_E_3_E, position numbers subscript) but containing the following amino acids _4_K_5_K_6_G_7_N were similarly recognized, but all peptides lacking more than three N-terminal amino acids of EGFRvIII, covering the complete sequences of the ECD, were not capable of being recognized by our diabody (data not shown). A more precise picture on which residues in the target sequence are crucial for antibody binding was obtained from the analysis of the binding capability to 15-mer peptides spanning the EGFRvIII N-terminal sequence and containing defined amino acid substitutions in every single position. Results showed that substitutions of specific target amino acid residues (with position numbers subscript) _4_K, _6_G, _7_N, _9_V, _10_V, and _11_T are not tolerated and completely disrupt the binding of our antibody (Figure 2D). By contrast, the substitution of residues _5_K, _8_Y, and _12_D do not disrupt the binding, except when these residues are substituted by proline (Figure 2D). Similarly, results on a set of peptides containing double alanine mutations indicated that mutations in positions 4–7 of EGFRvIII significantly decrease or totally disrupted the recognition of the target sequence by our antibody domains (data not shown). A corresponding epitope mapping analysis was performed with a diabody containing the parental anti-EGFRvIII domain Li3G30 and revealed very similar results, indicating that no major shift in the binding epitope was induced during the affinity maturation (data not shown).

### EGFRvIII-Binding Domains Are Unique in Their Specificity and Allow Distinctive Tumor Recognition

**Figure 3:**
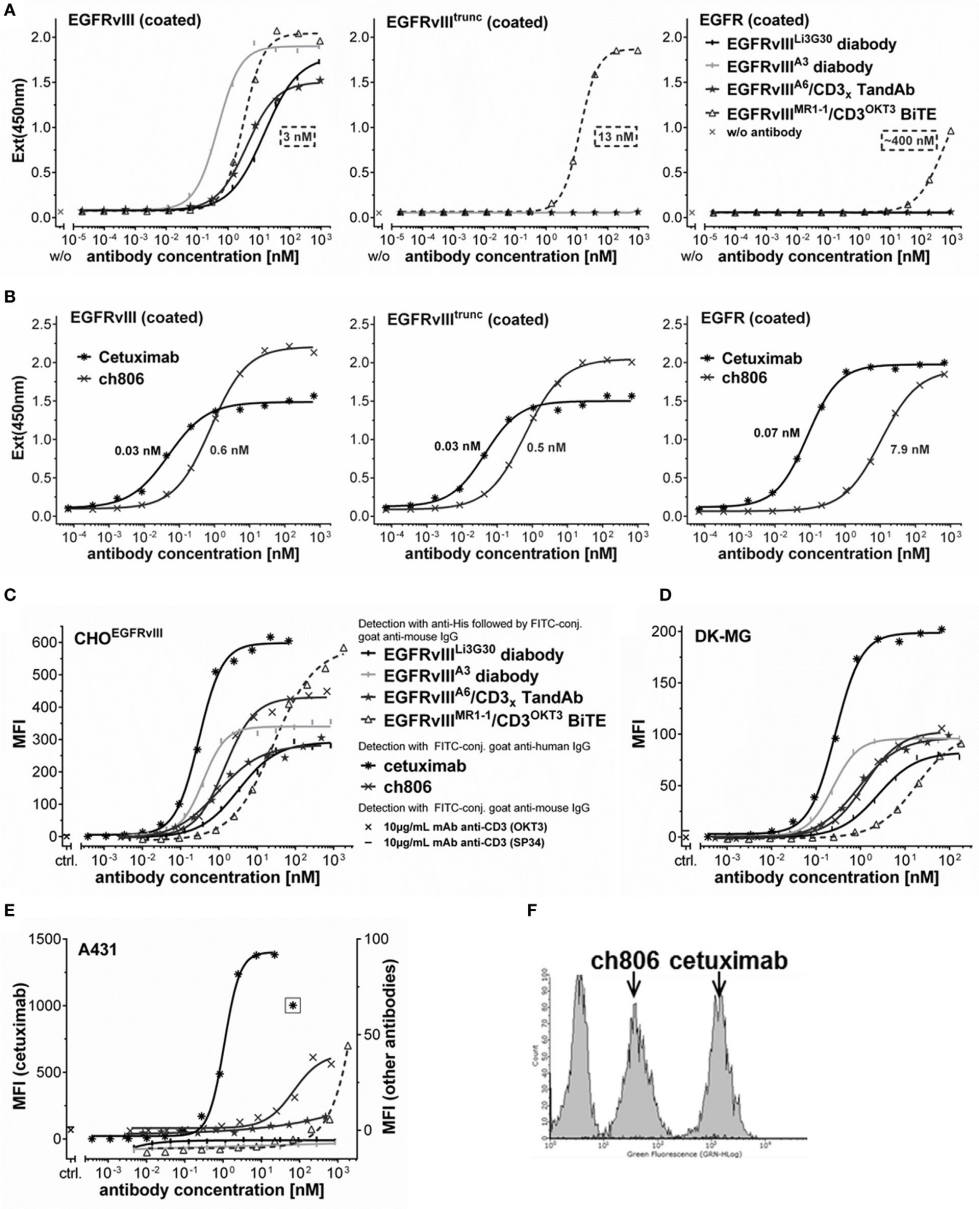
**Binding affinity and specificity for EGFRvIII antigen and on cells**. **(A,B)** Concentration-dependent binding of different His-tagged antibody fragments **(A)** or monoclonal antibodies **(B)** to recombinant EGFRvIII-Fc, EGFRvIII^trunc^-Fc, or EGFR-Fc coated in ELISA. *K*_D_ values indicated in the graphs were calculated with the equation one site binding (hyperbola), least squares (ordinary) fit using GraphPad Prism. **(C–E)** Concentration-dependent cell binding of different antibody constructs analyzed by sigmoidal dose–response/four parameter logistic fit: **(C)** CHO cells stably transfected to overexpress EGFRvIII, **(D)** DK-MG cells expressing EGFRvIII and EGFR, **(E)** A431 cells overexpressing EGFR (cetuximab staining signals on A431 cells due to high intensity are plotted on the separate left axis, signals of all other antibodies are plotted on the right axis with different scale). **(F)** FACS histogram overlay of A431 cells stained with saturating concentrations of ch806 or cetuximab or detection antibodies alone. MFI, mean fluorescence intensity, ctrl., control antibody staining at single dose of 10 µg/mL.

Recombinantly expressed EGFR and EGFRvIII antigen variants were used to further characterize the unique binding properties of our EGFRvIII-specific antibodies in comparison to other published EGFR- or EGFRvIII-targeting antibodies. Our EGFRvIII-specific diabodies or TandAbs containing either the low affinity (Li3G30) or affinity matured binding domains, specifically bound to EGFRvIII-Fc fusion antigen in ELISA in a concentration-dependent manner, but not to EGFR-Fc or a truncated EGFRvIII-Fc antigen variant lacking the six N-terminal amino acids of EGFRvIII (Figure 3A) at any of the tested concentrations, confirming that these antibodies require EGFRvIII’s N-terminal sequence _1_L_2_E_3_E_4_K_5_K_6_G for binding. The EGFRvIII^MR1-1^/CD3^OKT3^ BiTE prepared as described previously (33) showed binding to the EGFRvIII ECD antigen as well as to the truncated EGFRvIII variant, albeit with slightly reduced binding affinity (*K*_D_ values are indicated in Figure 3A), and at higher antibody concentrations of >1 μg/mL (~20 nM), it also bound to the immobilized EGFR ECD antigen (Figure 3A). These characteristics are similar to the binding properties of the antibody ch806 (34), which showed comparable binding to the EGFRvIII ECD antigen and its truncated form in ELISA, and recognized immobilized wild-type EGFR ECD antigen at higher concentrations (Figures 3A,B). These results are consistent with the published epitope for ch806, a loop structure which is exposed in EGFRvIII or in a transitional form of the non-mutated EGFR changing from the inactive tethered conformation to a ligand-bound active form (42). As expected, all three antigen variants were similarly well recognized by cetuximab (Figure 3B).

The binding properties and specificities of the different antibodies were also analyzed on EGFRvIII-expressing CHO or DK-MG cells, CHO cells overexpressing the truncated EGFRvIII antigen, or A431 cells expressing high levels of the wild-type EGFR (Figures 3C–E; data not shown). All of the tested antibodies showed a specific and concentration-dependent binding on the EGFRvIII-transfected CHO^EGFRvIII^ (Figure 3C) and endogenously EGFRvIII-expressing DK-MG (Figure 3D). Cetuximab recognizing both EGFRvIII and EGFR, allowed staining of the EGFRvIII- or EGFR-positive cells at lowest concentrations (Figures 3C–E), which is consistent with its high-binding affinity to different EGFR antigen variants (low *K*_D_ values between 0.03 and 0.07 nM) measured in ELISA (Figure 3B) and also published data by others (43). Among the tested EGFRvIII-specific antibodies, EGFRvIII/CD3 TandAbs or anti-EGFRvIII diabodies containing our affinity matured EGFRvIII-binding domains displayed the highest apparent affinity in EGFRvIII-positive cell binding, followed by antibodies containing the lower affinity anti-EGFRvIII domain Li3G30 or the EGFRvIII^MR1-1^/CD3^OKT3^ BiTE, which was the weakest EGFRvIII binder in our cell-binding assay (Figures 3C,D). High concentrations (>100 nM) of the latter also stained EGFRvIII-negative A431 cells (Figure 3E). Consistent with the results obtained in ELISA (Figures 3A–B) as well as published literature (42, 43), the antibody ch806 showed good and saturating binding on EGFRvIII-positive cells (Figures 3C,D) and only bound a low proportion of the amplified EGFR expressed on A431 cells (Figures 3E,F).

Improved binding to EGFRvIII-expressing cells of our EGFRvIII/CD3 TandAb antibodies was also analyzed in cell surface retention assays, which clearly demonstrated reduced dissociation for the TandAb antibodies containing our affinity optimized antibody domains (Figure S3 in Supplementary Material).

In summary, our EGFRvIII-targeting TandAbs and diabodies exhibit unique specificity and high affinity toward EGFRvIII with no residual binding to wild-type EGFR on both recombinant antigen and antigen-positive cells.

### Efficacy Tuning of EGFRvIII/CD3 TandAbs—Optimization of Target and Effector Cell-Binding Affinities

To identify an EGFRvIII-targeting cytotoxic antibody variant best suitable for therapeutic development, EGFRvIII-binding domains were used for the construction of different EGFRvIII-targeting, T-cell recruiting TandAbs which were extensively characterized regarding their binding to EGFRvIII and CD3 and ensuing cytotoxic properties.

**Figure 4:**
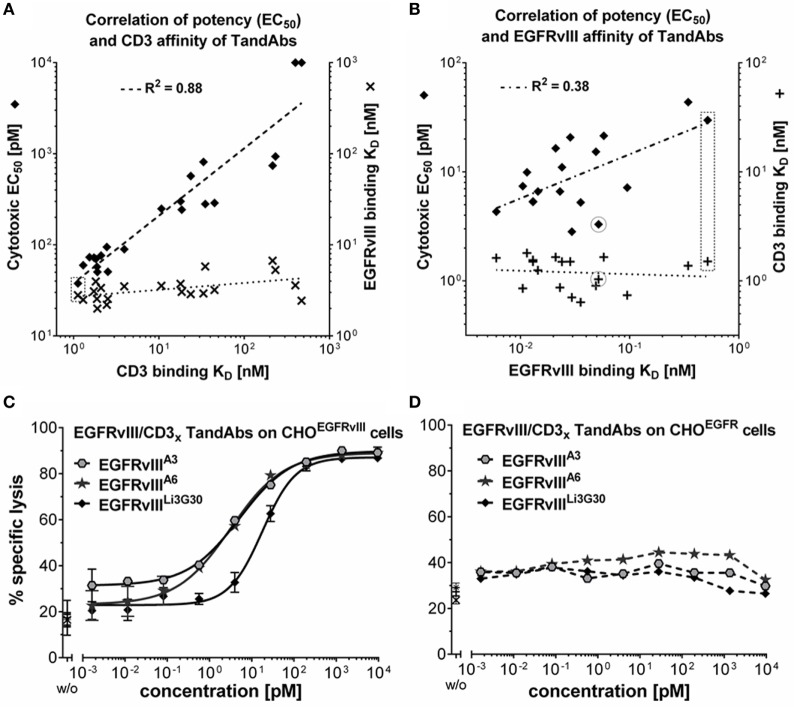
**Target and effector affinity-dependent T-cell-mediated cytotoxicity of EGFRvIII/CD3 tandem diabodies (TandAbs)**. **(A)** Illustration of the positive correlation of cytotoxic potency with CD3-binding affinities of EGFRvIII/CD3 TandAbs. More than 20 EGFRvIII/CD3 TandAbs all containing the identical EGFRvIII-binding domain (Li3G30) but different variants of CD3_x_-binding domains with a range of affinities for CD3 were characterized. CD3 binding was analyzed on CD3^+^-Jurkat cells, EGFRvIII binding on CHO^EGFRvIII^ cells, and cytotoxic potency in killing assays. EGFRvIII-binding *K*_D_ values (**x**) and cytotoxic EC_50_ values (♦) are plotted over the CD3-binding *K*_D_ values for each of the analyzed TandAbs. Log values were analyzed by linear regression showing the correlation of cytotoxic EC_50_ and CD3-binding *K*_D_ indicated by a dashed line; goodness of fit is shown as *R*^2^ value calculated with Graphpad Prism. **(B)** Illustration of the positive correlation of cytotoxic potency with EGFRvIII-binding affinities of EGFRvIII/CD3 TandAbs. 18 EGFRvIII/CD3 TandAbs all containing high-affinity human CD3-binding or human/cynomolgus cross-reactive CD3-binding domains combined with EGFRvIII-binding domains having a range of different affinities for EGFRvIII were characterized. CD3 binding was analyzed on CD3^+^-Jurkat cells, EGFRvIII-binding *K*_D_ values were measured in SPR and cytotoxic potency in killing assays. CD3-binding *K*_D_ values (**+)** and cytotoxic EC_50_ values (♦) are plotted over the EGFRvIII-binding *K*_D_ values for each of the analyzed TandAbs. Log values were analyzed by linear regression showing the correlation of cytotoxic EC_50_ and EGFRvIII-binding *K*_D_ indicated by a dashed line; *R*^2^ value was calculated using Graphpad Prism. Values of the EGFRvIII^Li3G30^/CD3_x_ TandAb are marked with a dashed box, and values of the selected EGFRvIII^A6^/CD3_x_ TandAb are circled. **(C)** Comparison of cytotoxic properties of EGFRvIII/CD3 TandAbs containing different anti-EGFRvIII domains A3, A6, or Li3G30 on CHO^EGFRvIII^. Lysis values obtained for a given antibody concentration were determined in duplicates and analyzed by sigmoidal dose–response/four parameter logistic fit analysis using Graphpad Prism software. **(D)** No specific lysis is mediated by these TandAbs on CHO^EGFR^ (FACS-based cytotoxicity assays with T-cells as effector cells at an E:T ratio of 5:1).

The EGFRvIII-binding domain Li3G30 was combined with a range of anti-CD3 domain variants having different affinities for CD3 (28). The resulting EGFRvIII/CD3 TandAbs were analyzed for CD3 binding on CD3^+^ Jurkat cells, for EGFRvIII-binding on CHO^EGFRvIII^ cells, as well as for cytotoxic potency in killing assays with EGFRvIII-positive target cells and T-cells as effector cells. Cytotoxic potency (EC_50_ values) showed an almost linear correlation with the *K*_D_ values for CD3 binding ranging from 1 to ~500 nM, whereas TandAbs showed only little variability in their binding on EGFRvIII-positive cells (Figure 4A). Among these low-affinity EGFRvIII-binding candidates, the highest *in vitro* cytotoxic activity was achieved by the EGFRvIII^Li3G30^/CD3_x_ TandAb that also exhibited the highest binding affinity for CD3 (*K*_D_ = 1.1 nM). Thus, we selected this high-affinity CD3-binding domain for further optimization of the target affinities in TandAbs. Different affinity matured EGFRvIII-binding Fv domains (Table 1) were combined in TandAbs with the previously selected high-affinity CD3-binding domain and analyzed for binding (Table 1) and cytotoxic properties (Table 2; Figures 4B,C). Whereas these TandAbs share very similar CD3-binding *K*_D_s of around 1 nM (Figure 4B; Figure S4 in Supplementary Material), the improvement in EGFRvIII-binding affinity for most of the TandAbs resulted in even stronger cytotoxic activity *in vitro* (Figure 4B). However, there were exceptions showing very strong cytotoxic activity despite intermediate binding affinity for EGFRvIII, like the TandAb EGFRvIII^A6^/CD3_x_ which was selected for further characterization based on its superior manufacturability profiles, such as expression, purification, and stability (Figure 1B; Figure S1 in Supplementary Material).

**Table 2:** **Cytotoxic potency of EGFRvIII/CD3 tandem diabody (TandAb) candidates**.

	EGFRvIII-binding Fv in TandAb	Effector binding Fv in TandAb	No. of assays	Cytotoxic potency (EC_50_) mean of *n* ≥ 4 (pM)	SD
Parental	Li3G30	CD3_x_	10	29.8	±19.7
Affinity matured	A3	CD3_x_	7	4.3	±3.3
	A4	CD3_x_	4	7.4	±4.5
	A6	CD3_x_	6	3.3	±1.3
	A7	CD3_x_	4	11.0	±2.6
	A8	CD3_x_	4	7.2	±1.3
	A9	CD3_x_	4	5.3	±1.9

*Individual results were measured in FACS-based cytotoxicity assay on CHO^EGFRvIII^ target cells*.

### EGFRvIII/CD3 TandAbs Efficiently and Specifically Engage T-Cells Enabling Potent Killing of EGFRvIII-Positive Target Cells *In Vitro* and *In Vivo*

Affinity optimized EGFRvIII/CD3 TandAbs were most potent in killing assays, displaying cytotoxicity toward EGFRvIII-expressing CHO, human DK-MG, or F98 glioma cells with EC_50_ values in the range of 2–10 pM (Table 2; Figure 4C; Figure S5 in Supplementary Material). *In vitro* cytotoxic potency of EGFRvIII/CD3 TandAbs was clearly superior to that of a previously published EGFRvIII^MR1-1^/CD3^OKT3^ BiTE (33, 41) (Figure S5 in Supplementary Material).

**Figure 5:**
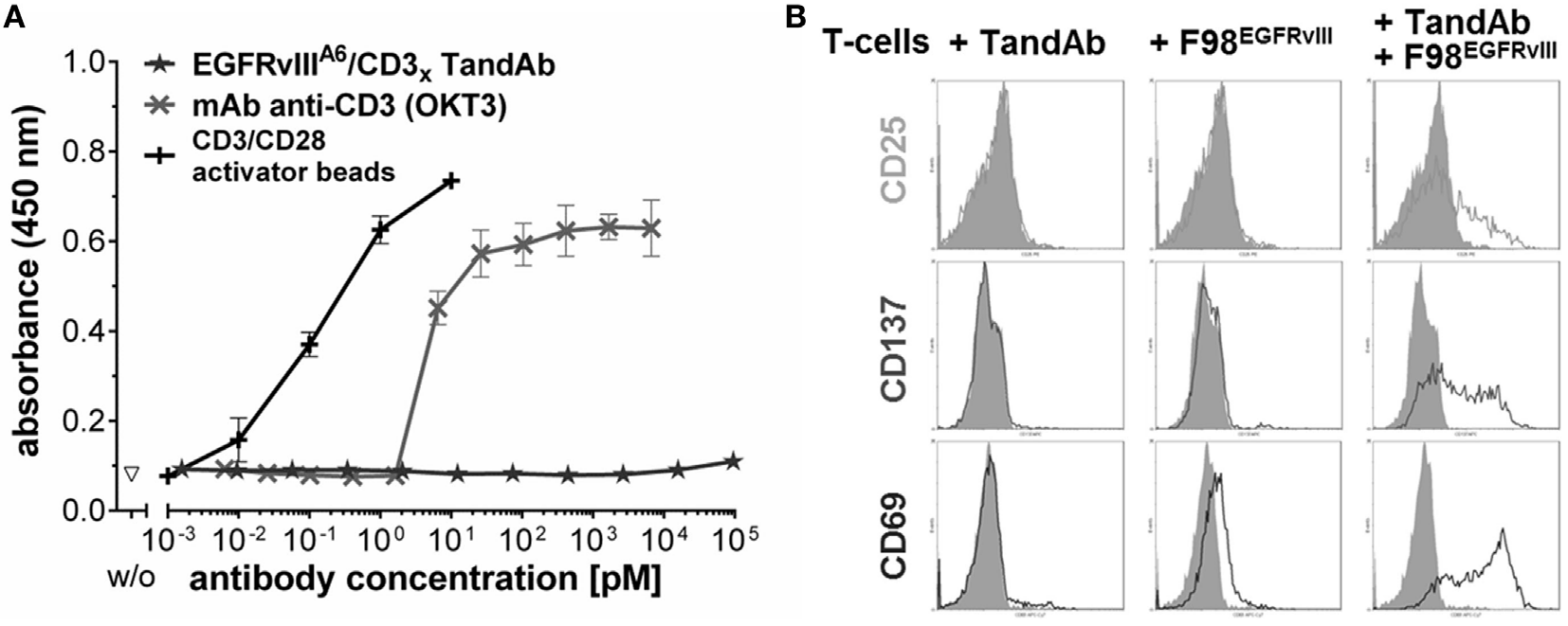
**EGFRvIII/CD3 tandem diabody (TandAb) does not facilitate activation of human T-cells in the absence of EGFRvIII-positive target cells**. **(A)** No PBMC proliferation is induced by EGFRvIII^A6^/CD3_x_ TandAb despite bivalent binding sites for CD3 after cultivation for 5 days and analyzed by BrdU incorporation assay. Control antibody OKT3 induces proliferation. Control cells were cultured in the absence of antibodies (w/o). Mean absorbance, and SD of triplicates, measured at 450 nm are plotted. **(B)** T-cell activation marker analysis by FACS: primary human T-cells were cultured for 24 h with or without 10 µg/mL of EGFRvIII^Li3G30^/CD3_x_ TandAb in the presence or absence of F98^EGFRvIII^ target cells at an E:T ratio of 1:1 before T-cells were analyzed for expression of the activation markers by flow cytometry. As a control, cells were cultured in the absence of antibodies and in the absence of target cells (gray histograms). Activation markers are induced on T-cells only when both, target-positive cells and the TandAb are present.

No cytotoxicity was observed on EGFR-positive cells or EGFRvIII-negative cells demonstrating the high selectivity of EGFRvIII/CD3 TandAbs for the tumor-specific EGFRvIII (Figure 4D). High-affinity binding to CD3 was beneficial for efficacious T-cell recruitment and T-cell-mediated target cell killing as shown by the correlation of the apparent affinity to CD3-positive cells and cytotoxic potency of EGFRvIII/CD3 TandAbs (Figure 4). Importantly, in the absence of EGFRvIII-positive target cells *in vitro*, TandAbs did not elicit T-cell activation, as demonstrated by their lack of proliferation (Figure 5A) and induction of activation markers (Figure 5B). For efficacious activation, T-cells required both EGFRvIII-positive target cells and EGFRvIII/CD3 TandAb (Figure 5B). Together with the high specificity for EGFRvIII and the absence of off-target activity, this contributes to a good safety profile of EGFRvIII/CD3 TandAbs.

**Figure 6:**
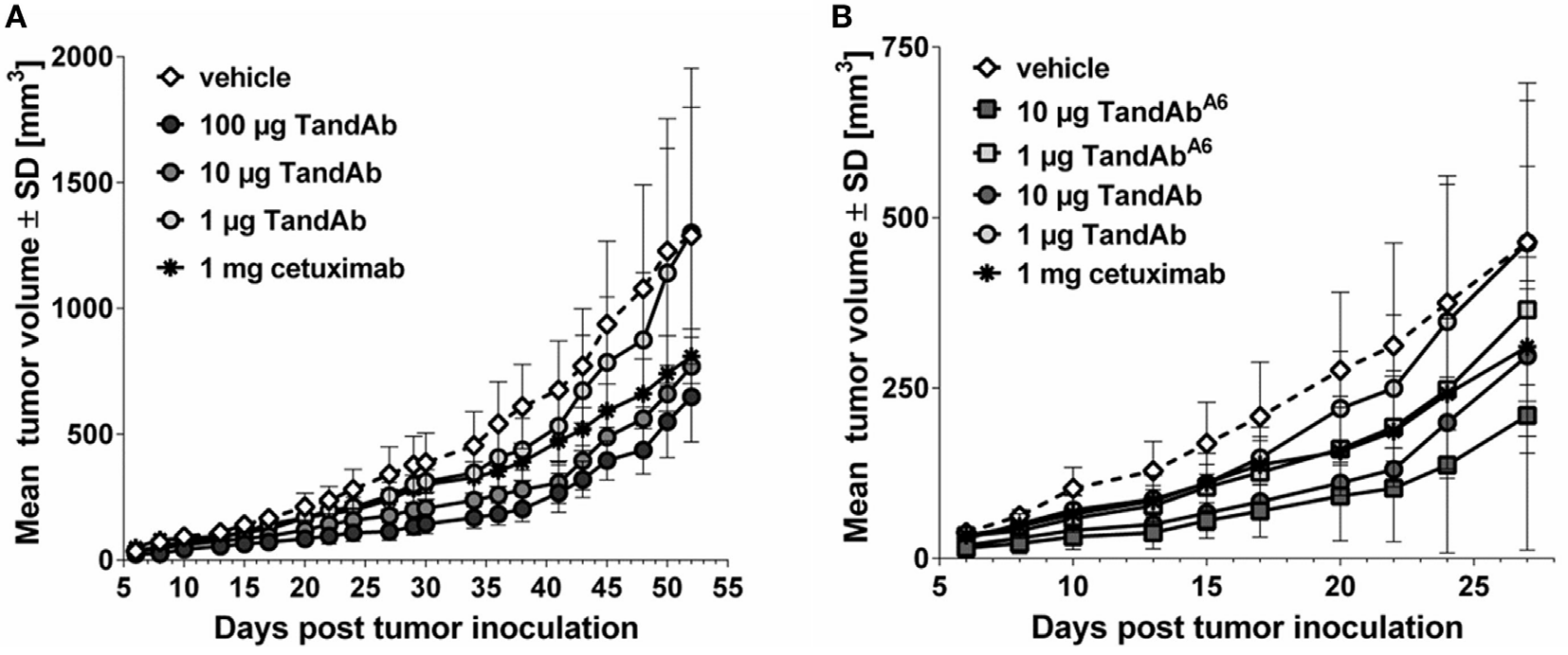
**EGFRvIII-binding antibodies reduce the growth of F98^EGFRvIII^ xenograft tumors in mice**. NOD/scid mice were xenotransplanted by subcutaneous injection of 4 × 10^6^ F98^EGFRvIII^ cells mixed with 1 × 10^7^ human PBMC on day 0. Tandem diabody (TandAb) or vehicle was dosed intravenously as indicated 2 h post-tumor cell inoculation and on four subsequent days. Cetuximab was dosed intraperitoneal twice a week with 1 mg starting on day 0. **(A)** Dose-dependent tumor growth inhibition by EGFRvIII^Li3G30^/CD3_x_ TandAb (containing the non-affinity optimized EGFRvIII-binding domain Li3G30). Statistical analysis (two-way repeated measures ANOVA with Bonferroni posttests) was performed and showed significant differences between the vehicle group and the 100-µg TandAb-treated group on day 38, day 41 (*p* < 0.05), day 43, and day 45 (*p* < 0.01). **(B)** Comparison of subcutaneous F98^EGFRvIII^ xenograft tumor growth retardation by EGFRvIII^Li3G30^/CD3_x_ TandAb and EGFRvIII^A6^/CD3_x_ TandAb containing an affinity matured anti-EGFRvIII domain (A6) at two dose levels each. This study was terminated after 27 days due to frequent tumor growth into the abdominal cavity and adjacent organs. Statistical analysis was performed and showed significant differences between the vehicle group and the 10-µg TandAb A6-treated group on days 6–22 (*p* < 0.05), or the 10-µg TandAb-treated group on days 8–20 (*p* < 0.05).

Efficacy of EGFRvIII/CD3 TandAbs *in vivo* was demonstrated in an exploratory subcutaneous xenograft tumor model using immunodeficient mice reconstituted with human PBMC as effector cells. Treatment of these mice with EGFRvIII/CD3 TandAb for five consecutive days induced a dose-dependent growth retardation of subcutaneously growing F98^EGFRvIII^ tumors with a TandAb candidate containing the low-affinity EGFRvIII-binding domain Li3G30 (Figure 6A). At dose levels of 10 µg/injection and 100 µg/injection of the EGFRvIII/CD3 TandAb, statistically significant tumor growth inhibition was measured relative to the vehicle group from days 6 to 45. The tumor inhibition achieved with the anti-EGFR antibody cetuximab, though not statistically significant, was comparable to or less pronounced than EGFRvIII/CD3 TandAb, despite the fact that cetuximab was administered at a 10- to 100-fold higher dose (1 mg/injection) twice weekly (Figure 6A). In a follow-up study, a TandAb containing the affinity matured anti-EGFRvIII domain A6 was investigated in the same F98^EGFRvIII^ model and also demonstrated tumor growth inhibition (Figure 6B). However, due to frequent tumor outgrowth into the abdominal cavity and adjacent organs, this follow-up study needed to be terminated prematurely and thus failed to confirm *in vivo* the superiority of the affinity optimized TandAb candidate. Several other models including orthotopic settings are currently in planning.

In summary, our highly selective EGFRvIII/CD3 TandAbs are potent new drug candidates mediating killing of EGFRvIII-positive tumor cells by efficacious T-cells engagement.

## Discussion

EGFRvIII is a well-validated target for therapeutic development, offering the potential of specificity in combination with efficacy and safety. Therefore, many different antibodies against this attractive target have been generated already more than two decades ago using rabbits (8), hybridoma (11, 44–46), and/or phage display technology (47, 48). Almost all antibodies were generated by immunization using synthetic peptides spanning the unique junctional sequence of the N-terminus of EGFRvIII. Different modes of actions have been applied to improve these antibodies’ therapeutic efficacies *in vitro* and *in vivo*. Ranging from naked antibodies (49–52), over immunotoxins (48, 53, 54), to radio- (55, 56) or antibody drug conjugates (ADCs) (17, 57). In addition, for several antibodies, blocking of the cell-signaling pathway of EGFRvIII has been demonstrated (58). Despite numerous reports on efficacy *in vitro* or preclinical *in vivo* data showing proof-of-concept, very little therapeutic progress has been reported with regard to EGFRvIII targeting. Thus far only two EGFRvIII-specific ADC derivatives are currently in clinical trials: ABT-414, an ABT-806 monomethyl auristatin F conjugate has advanced to phase II clinical trials (16, 57) after its naked, chimeric parental antibody showed excellent targeting of tumor sites in all patients (51); AMG 595, composed of the maytansinoid DM1 attached to a highly selective anti-EGFRvIII antibody has completed a Phase 1 study (17). However, as with other ADC approaches, serious side effects may be triggered by dosing, drug design, or off-target binding (21). The latter point will be most relevant for antibodies that are not exclusively specific for EGFRvIII, such as the antibody ABT-806, which has been reported to also bind to the activated wild-type EGFR (42). Dermal toxicities commonly observed during therapy with mAbs targeting the wild-type EGFR should not be induced when targeting the tumor-restricted mutant EGFRvIII. More recently, the concept of T-cell engagement has been added to the armamentarium of EGFRvIII-mediated tumor targeting with a BiTE (33, 41). At the same time, approaches to develop wild-type EGFR-targeting T-cell engagers did not proceed, likely due to the high risk of toxicities based on the broad expression of EGFR in healthy tissues (59). Moreover, several approaches using EGFRvIII-targeting CAR T-cells have entered clinical investigation in patients with GBM (22, 60–62). Although functionally effective, CAR T-cell technology has its limitations due to the high technical burden of this personalized medicine. By contrast, bispecific antibodies such as T-cell engagers could be equally effective and more universally applied to patients. Here, we demonstrate a new strategy to target EGFRvIII-positive tumors with a bispecific tetravalent TandAb format (24), which is thus far superior in specificity, affinity, and *in vitro* efficacy compared to other published EGFRvIII antibodies and formats.

We identified a new, highly EGFRvIII-specific variable domain, Li3G30, by phage display screening and selection with an initial affinity of 17.5 nM from a naïve fully human antibody library (35, 36). This monovalent affinity falls within the range of published anti-EGFRvIII affinities ranging from 23 nM for other monovalent antibody constructs down to 0.74 nM for bivalent IgG or affinity improved antibody constructs (17, 32, 33, 48). In order to potentially enhance efficacy in a TandAb molecule, the Li3G30 clone was affinity matured to reach sub-nanomolar affinities. The potential of the applied AbAccel™ technology for affinity maturation has previously been demonstrated (28). Even in this challenging project, where the increase in affinity to the deletion mutant EGFRvIII should not lead to a gain in binding to wild-type EGFR, affinity improvements of more than 50-fold were reached. Thus far the monovalent affinities of 0.16 and 0.25 nM of clones A4 and A3 demonstrate one of the best affinities among published anti-EGFRvIII domains—even superior to the affinity improved EGFRvIII-recognizing domain MR1-1 that has also been subjected to affinity maturation from 24 nM (MR1) (48) down to 1.5 nM (32).

In order to differentiate our domain from other published EGFRvIII-binding domains, a detailed epitope analysis was performed. As mentioned earlier, in most cases, synthetic peptides surrounding the junction have been used to produce monoclonal and polyclonal antibodies, except for antibody ch806, a mAb selected after immunization with cells expressing EGFRvIII (34). For direct comparison, MR1-1 [as EGFRvIII/CD3 BiTE (33)] and ch806 were included in our study. In contrast to the fully human antibody sequence of our Li3G30 domain and its affinity matured derivatives, the MR1-1 domain is still a murine antibody and may cause neutralizing antibodies in patients. Others have shown a humanized variant of MR1-1 but thus far not in bispecific format (63). The results showed that binding of our EGFRvIII-specific domain Li3G30 and the affinity matured clones was clearly dependent on the presence of the N-terminal EGFRvIII-specific deletion/fusion sequence (9). By contrast, MR1-1 and ch806 also reacted with full-length EGFR as previously shown (64), which indicate a different, less specific epitope. This is most likely due to the nature of the peptide used for the generation of MR1 covering the first 13 amino acids including the unique junction of EGFRvIII (48) and also sequences recognizable in the wild-type EGFR molecule. The observed strong non-specific reaction of MR1-1 in Western Blots (Figure 2B) could also be due to the denaturing conditions, as an EGFRvIII-specific conformational epitope was also postulated for the MR1-1 antibody (65).

As the epitope for Li3G30 and its optimized derivatives seems less dependent on the conformation, further analyses were carried out on 15-mer peptides with defined amino acid substitutions. Results show that crucial amino acids reside within positions 4–11 including the EGFRvIII-specific glycine residue at position 6. The latter amino acid seems less important for MR1-1 binding (65), which again points out to a different epitope of these antibodies. It cannot be ruled out that other EGFRvIII-specific antibodies generated by peptide immunization (11, 44) recognize a similar epitope. However, all these findings still support our strategy to use phage display technology in combination with fully human antibody libraries to generate unique anti-EGFRvIII-specific antibodies with new epitope specificities that may have not been selected by immunizations.

An antibody variant of MR1-1, called RAb^DMvIII^ containing two amino acid mutations in CDRH2 and CDRH3 and the scFv fused to human IgG Fc revealed better specificity for EGFRvIII and less cross-reactivity with the wild-type receptor (66), yet the reported dissociation constant for RAb^DMvIII^ of 886 nM indicates much weaker binding than that of the affinity improved EGFRvIII-binding antibody constructs generated by us and others. These findings also show that sterical aspects of the EGFRvIII-binding antibody domains’ arrangement relative to the antigen strongly influence binding strength. While the bivalent binding sites in the diabody or TandAb format are clearly in favor of binding by improved avidity relative to monovalent scFv (Table 1), this does not simply translate into any other antibody format containing one or two MR1-1-derived binding sites (32, 33, 66).

The EGFRvIII-specific antibodies were further characterized in ELISA and FACS in comparison to MR1-1, ch806, and cetuximab. As already demonstrated in Western Blot experiments, Li3G30 and its matured clone A3 maintained its superior specificity toward EGFRvIII in ELISA. Despite non-denaturing conditions applied in this ELISA, cross-reactivity against EGFR could still be demonstrated for MR1-1, at concentrations above 400 nM. Again, this clearly indicates a different epitope for Li3G30 and greater specificity toward EGFRvIII. The cross-reactivity of antibody ch806 to EGFR is consistent with its published epitope, a loop structure, which is constitutively exposed in EGFRvIII but only transiently in the wild-type EGFR during ligand binding (42). Finally, EGFRvIII specificity could be confirmed on cells where Li3G30 and its affinity matured antibody derivatives bound exclusively to EGFRvIII-positive cells, but not to highly EGFR-positive A431 (Figure 3E).

Antibody-mediated engagement of T-cells was previously shown to enable very powerful anti-cancer responses (67). By using antibody engineering technologies, it has become possible to create a plethora of bispecific antibody formats with different variable domains (68, 69), which can also be used to engage cytotoxic immune cells to attack tumors. Through mono- or multivalent bispecific designs, these constructs tether T-cells and cancer cells, resulting in localized and specific T-cell activation and subsequent tumor lysis. Two examples of the leading bispecific antibody formats consisting of variable domains are BiTEs and TandAbs, both clinically validated platform molecules (26, 27, 70). The BiTE format was also used to target EGFRvIII-positive cells with very promising *in vitro* and *in vivo* efficacy (33). For direct comparison with the TandAb format, we have generated this EGFRvIII-specific BiTE according to its published sequences for MR1-1 and OKT3 (31, 32, 48). The EGFRvIII-specific BiTE revealed affinities of 1.5 and 6.5 nM for EGFRvIII (MR1-1) and CD3 (OKT3), respectively (33), which translated into EC_50_ values between 10 and 35 pM as shown in our cytotoxicity assays on DK-MG cells (Figure S5 in Supplementary Material). In the same experiment, the EGFRvIII-specific TandAb showed 10-fold lower EC_50_ most likely due to its favorable tetravalent domain arrangement and higher binding affinities for EGFRvIII on the tumor cell and CD3 on the T-cell. In fact, we demonstrated a positive correlation of cytotoxic potency with increasing CD3-binding affinities, as illustrated for EGFRvIII/CD3 TandAbs (Figure 4). *In vitro* cytotoxicity assays on F98^EGFRvIII^ cells revealed slightly higher EC_50_ values compared to those obtained on DK-MG or CHO^EGFRvIII^ as targets, but a similar ranking of the different bispecific EGFRvIII/CD3-targeting drug candidates (Figure S5C in Supplementary Material).

An important requirement for clinical development is the manufacturability of the product. As TandAbs are already in clinical trials (26, 27, 70), important parameters such as stability and productivity of this drug class are well established. Li3G30 and its affinity optimized derivatives combined with different anti-CD3 effector cell-binding domains in TandAbs showed shake-flask expression titers between 25 and 250 mg/L, with the TandAb containing anti-EGFRvIII clone A6 in combination with the human and cynomolgus cross-reactive anti-CD3 domain shown to be the best regarding expression. T-cell-engaging bispecific antibodies are clinically used at nanograms per kilogram to micrograms per kilogram doses, which is far below the application levels of marketed therapeutic mAbs that are dosed at milligrams per kilogram. Thus, it can already be anticipated that productivity after stable cell line generation will be feasible for the lead candidate. The TandAb containing anti-EGFRvIII clone A6 also demonstrated very good stability in an accelerated stability study at 40°C with an incubation period of 7 days. This is in line with other T-cell recruiting TandAbs currently in clinical development (26, 28). Given the fact that TandAbs can be produced cheaply and are available “off-the-shelf,” this technology has an advantage over CAR T-cell therapies, which need to overcome expensive manufacturing and logistics before qualifying for broad clinical application.

In a preliminary *in vivo* pilot study, our anti-EGFRvIII-specific TandAbs showed a robust dose-dependent growth inhibition of EGFRvIII-expressing subcutaneous xenograft tumors in NOD/scid mice reconstituted with human PBMCs. In comparison to cetuximab, which was used as a system suitability control and administered in at least 10-fold higher doses according to a previously published protocol (59), the tumor growth inhibition by EGFRvIII/CD3 TandAb was more pronounced. Several other EGFRvIII-binding antibodies have already been tested in xenograft mouse models, either in subcutaneous or orthotopic settings. The antibodies were either used alone (43, 50, 52), in combination with chemotherapeutics (71), as conjugates (17, 72) or bispecifics (33), which makes an *in vivo* comparison of these anti-EGFRvIII domains difficult. Another limitation is the use of different EGFRvIII-positive cell lines as xenografts. Almost all of them had to be transfected with EGFRvIII to maintain stable target expression that would otherwise be lost under *in vitro* cell culture conditions (73, 74), which might contest the relevance of such xenograft models. Our follow-up study needed to be terminated prematurely and thus failed to confirm *in vivo* the superiority of the affinity optimized TandAb candidate. It is also important to note that a subcutaneous xenograft is not an ideal model, for example, glioblastoma. In this case, orthotopic or in particular intracranial models for GBM with a humanized immune effector cell background (33) will be more meaningful, but also much more difficult and laborious than the presented model. Such models are currently under investigation for further *in vivo* proof-of-concept studies with our lead anti-EGFRvIII TandAb.

Beyond evidence of dose-dependent tumor growth inhibition *in vivo*, it is important to note that the presented fully human EGFRvIII-targeting TandAbs differentiates from all previously described EGFRvIII-targeting antibody therapeutics in their exclusive specificity for the tumor-restricted EGFRvIII neoepitope. This provides the unique opportunity to harness the cytotoxic capacity of T-cells to selectively target cancer cells without harming EGFR-positive healthy tissues, thereby EGFRvIII/CD3 TandAb therapeutics might offer superior safety and patient convenience, while achieving highly efficacious tumor control.

## Ethics Statement

All animal experiments were performed at the Heidelberg Pharma GmbH animal facility. The study and the protocol were reviewed and approved by Heidelberg Pharma’s “Tierschutzausschuss” (ethical committee) to guarantee ethical standards which are according to the German animal welfare law based on EU-regulation (2010/63 EU). Accreditation of tumor studies was done by the mid-level local authority (Regierungspräsidium Karlsruhe), supervised by the lower-level local authority (Veterinäramt Wiesloch), regulated by European Union law and directives as well as German national law.

## Author Contributions

KE designed, conducted, and analyzed experiments, particularly cell line generation, protein expression, and ELISAs. UR coordinated, performed, and analyzed cell-based *in vitro* assays. IF carried out all molecular biology work. SK was involved in the design and coordination of the *in vivo* models. MW was responsible for purification and biochemical characterization of the proteins. TG contributed to the design of certain experiments. VM coordinated the selection of scFv and the affinity maturation. EZ supervised the project and contributed to design of experiments before 2015. MTesar took over the project leadership and assisted in writing the manuscript. MTreder was responsible for the project and its strategy after 2015.

## Conflict of Interest Statement

KE, UR, IF, SK, MW, TG, MTesar, and MTreder are employees and hold stock options of Affimed. EZ was employee and consultant of Affimed. KE, UR, IF, SK, VM, and EZ are co-inventors on patent applications on antibody binding sites specific for EGFRvIII.
