# Supplementary material for: Highly Specific and Effective Targeting of EGFRvIII-Positive Tumors with TandAb Antibodies
Source: Front Oncol. 2017 May 19;7:100. doi: 10.3389/fonc.2017.00100 (PMC5442391; doi:10.3389/fonc.2017.00100)
Supplement: Supplementary file 1 [file Data_Sheet_1.DOCX]

Supplementary Material

Highly specific and effective targeting of EGFRvIII-positive tumors with TandAb antibodies

Kristina Ellwanger, Uwe Reusch, Ivica Fucek, Stefan Knackmuss, Michael Weichel, Thorsten Gantke, Vera Molkenthin, Eugene A Zhukovsky, Michael Tesar*, Martin Treder

*** Correspondence:**Corresponding Author: Michael Tesar
M.Tesar@affimed.com

# Supplementary Figures and Tables

## Supplementary Figure 1

**A B**

**

**


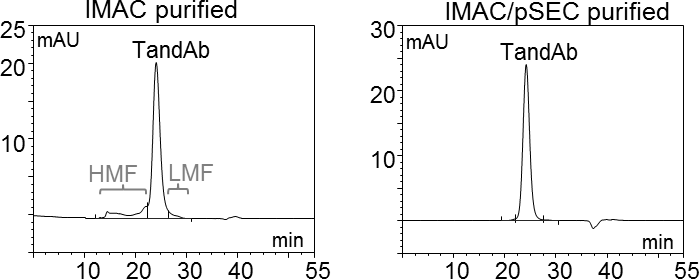


**C**

**

**

**Supplementary Figure 1.** **Production, purification and stability of EGFRvIII^A6^/CD3_x_ TandAb. (A)** Exemplary data from research stage production of EGFRvIII^A6^/CD3_x_ TandAb expressed in a 10‑day fed-batch shake-flask culture of stably-transfected CHO cells, which grow to high cell densities (●), maintain high viability (**o**), and secrete the TandAb product into the cell culture supernatant (CCS) (■). **(B)** Exemplary analytical SEC data from IMAC purified EGFRvIII^A6^/CD3_x_ TandAb or material further purified by preparative size exclusion chromatography (SEC). After IMAC, minor fractions of high molecular weight forms (HMF) appear as peak areas with shorter retention time consistent with an apparent molecular mass of ≥200 kDa or very minor fractions of low molecular weight forms (LMF) appearing as peaks with higher retention times consistent with molecular weights of <60 kDa. (C) Molecular forms of the purified EGFRvIII^A6^/CD3_x_ TandAb in acetate buffer (non-formulated) were analyzed by analytical SEC after incubation at 4°C, 37°C or 40°C for the indicated numbers of days (d).

## Supplementary Figure 2

**A B C**

**
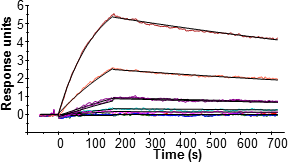

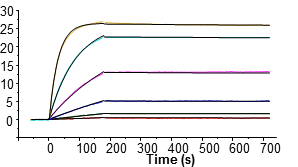

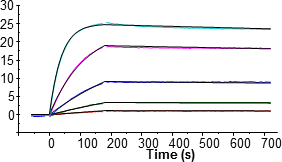
**

**D E F**

**
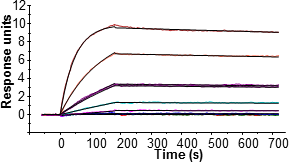

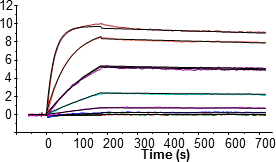

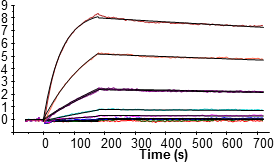
**

**G**

**
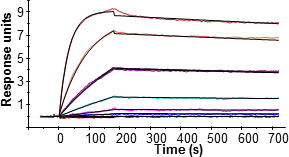
**

**Supplementary Figure 2. SPR multi-cycle kinetics measurement of binding of different EGFRvIII/CD3_x_ TandAb antibodies to recombinant EGFRvIII-Fc antigen.** The TandAb measured in **(A)** contains the low affinity parental EGFRvIII-binding domain Li3G30. The other panels show binding sensorgrams for TandAbs containing affinity matured EGFRvIII-binding domains **(B)** A3, **(C)** A4, **(D)** A6, **(E)** A7, **(F)** A8, **(G)** A9. Sensorgrams show the kinetics of surface plasmon resonance (SPR) response units (RU) measured over time at different concentration levels of the antibodies. Dilution factor was 3 for all dilution series. TandAb antibodies in (A) and (D-G) were measured at 8 concentrations starting at 9 nM, TandAb in (B) was analyzed at 6 concentration levels starting at 10 nM, for the TandAb shown in (C) 5 concentrations starting at 3.3 nM were analyzed. Association is measured for 180 sec, dissociation is measured thereafter. Data was analyzed using a 1:1 binding model.

## Supplementary Figure 3





**Supplementary Figure 3. Retention of different EGFRvIII/CD3 TandAb antibodies on the surface of EGFRvIII expressing CHO cells.** After binding to target antigen positive cells, TandAb antibodies containing the affinity matured EGFRvIII-binding domains A3, A4, A6 or A7 show clearly improved retention (slower dissociation) relative to the TandAb containing the parental EGFRvIII-binding domain Li3G30.

## Supplementary Figure 4

**A**





**B**





**Supplementary Figure 4. Binding of different TandAbs to recombinant CD3 antigen or primary T-cells. (A)** Binding of different EGFRvIII/CD3_x_ targeting TandAbs to coated recombinant CD3ε in ELISA is not affected by different EGFRvIII-binding moieties. **(B)** Binding of different antibodies to CD3 on T-cells analyzed by flow cytometry. All tested EGFRvIII/CD3_x_ TandAbs show very similar binding to CD3 on T-cells. ctrl.: control antibody staining at a single dose of 10 µg/ml.

## Supplementary Figure 5

**A**





**B**





**C**





**Supplementary Figure 5. Comparison of cytotoxic activity of EGFRvIII/CD3 TandAb and BiTE or ADCC mediated by comparator anti-EGFRvIII IgG and on different target cell lines. (A)** EC_50_ values obtained for three different EGFRvIII-targeting antibodies in n=3 independent cytotoxicity assay with DK-MG as targets and PBMC as effector cells (1x10^4^ targets/well; E:T ratio 25:1; 4 h incubation). Mean values are shown as a line. **(B)** Exemplary killing curves from one of these cytotoxicity assay with DK-MG as targets. **(C)** Exemplary killing curves from a cytotoxicity assay with F98^EGFRvIII^ as target cells and PBMC as effector cells (1x10^4^ targets/well; E:T ratio 50:1; 4 h incubation).
